# The Prognostic Role of STAT5B Across Cancer Types and Comparative Analysis with STAT5A: A Systematic Review

**DOI:** 10.3390/biom15111503

**Published:** 2025-10-24

**Authors:** Christine Maninang, Jinghong Li, Willis X. Li

**Affiliations:** Department of Medicine, University of California, La Jolla, San Diego, CA 92037, USA

**Keywords:** STAT5, systematic review, meta-analysis, cancer

## Abstract

Background: The signal transducer and activator of transcription 5 (STAT5) proteins, STAT5A and STAT5B, are highly homologous transcription factors with distinct roles in cancer biology. While STAT5A has been characterized as a context-dependent modulator of tumor progression, the prognostic significance of STAT5B remains less clear. Here, we conducted a systematic meta-analysis of STAT5B to evaluate its association with overall survival across cancers and to compare its prognostic role with that of STAT5A, as reported previously. Methods: Microarray datasets from the Prognoscan database were analyzed for STAT5B expression and overall survival. Hazard ratios (HRs) were estimated using Cox proportional hazards models, and results from 42 datasets were synthesized by meta-analysis. Subgroup analyses were performed by cancer type, and heterogeneity was assessed using Cochran’s Q Test and I^2^ statistics. Results: Pooled analysis showed that high STAT5B expression was significantly associated with favorable overall survival (lnHR = −0.4009; 95% CI: −0.6007 to −0.2011; *p* < 0.0001), albeit with notable heterogeneity (I^2^ = 64%). Subgroup analyses indicated that STAT5B was particularly protective in lung cancers (lnHR = −0.5170; *p* = 0.0042) and hematologic malignancies (lnHR = −0.6988; *p* < 0.0001). In contrast, STAT5A demonstrated divergent effects, conferring favorable survival in breast cancer but poorer outcomes in hematologic cancers. Conclusions: Elevated STAT5B expression is associated with improved survival in multiple cancers, supporting a potential tumor-suppressive role distinct from STAT5A. These findings underscore the importance of isoform-specific STAT5 evaluation in cancer prognosis and suggest that STAT5B may serve as a potential biomarker and therapeutic target.

## 1. Introduction

Cancer continues to be one of the most significant challenges to global public health, accounting for nearly 10 million deaths annually and imposing a substantial burden on healthcare systems worldwide. Despite advancements in early detection and therapeutic interventions, the heterogeneity of malignant diseases and the emergence of treatment resistance underscore the urgent need for novel prognostic biomarkers and targeted therapies. Among the molecular pathways implicated in oncogenesis, the Signal Transducer and Activator of Transcription (STAT) family of transcription factors has garnered considerable attention due to its central role in regulating cell proliferation, survival, and immune responses. In particular, STAT5, which exists as two closely related isoforms, STAT5A and STAT5B, has emerged as a critical player in cancer biology, exhibiting complex and context-dependent functions that remain incompletely understood [1,2,3,4]. To clarify the prognostic significance of STAT5 isoforms, we previously performed a systematic meta-analysis of STAT5A across cancer types using publicly available microarray datasets [5], and in the present study, we extend this approach to STAT5B, enabling a direct comparison between the two isoforms.

STAT5A and STAT5B share approximately 90% sequence homology and are encoded by genes located in tandem on human chromosome 17q21.2, separated by a mere 12.5 kb [1,2,3,4]. This genomic arrangement, conserved across mammals, suggests evolutionary co-regulation while allowing for independent transcriptional control [6,7]. Both isoforms possess similar promoter regions, including γ-interferon activation sites (GAS) and binding motifs for transcription factors such as SP1, C/EBPβ, and glucocorticoid receptors [3]. Despite these structural similarities, STAT5A and STAT5B exhibit divergent expression patterns and functional roles across tissues and malignancies [8]. For instance, growth hormone (GH) preferentially activates STAT5B in hepatocytes, where it regulates metabolic and growth-related genes [9], whereas prolactin predominantly signals through STAT5A in mammary epithelium, influencing lactation and breast tissue homeostasis [10]. Such stimulus- and tissue-specific regulation highlights the non-redundant biological roles of these isoforms, even within shared signaling pathways.

The functional dichotomy of STAT5 isoforms extends to their roles in cancer, where they can act as either oncogenes or tumor suppressors, depending on the cellular context. In our previous work, we systematically analyzed STAT5A expression across 42 cancer types, revealing its dualistic prognostic impact: elevated STAT5A levels were associated with favorable survival outcomes in breast and bladder carcinomas but with a poorer prognosis in hematologic malignancies [5]. These findings underscored STAT5A’s dual nature as both an oncogene and tumor suppressor, possibly influenced by tissue-specific signaling networks and interactions with other STAT family members, particularly STAT3, in shaping STAT5A’s role in cancer progression. For instance, STAT3 and STAT5 exhibit reciprocal and sometimes opposing effects in breast cancer on gene expression and cellular behavior [11,12]. While both are involved in mammary gland development and can be activated in breast cancer, STAT5 tends to promote differentiation and survival, while STAT3 can be associated with proliferation and survival, but also apoptosis. Importantly, in cases where both are activated, STAT5 often exerts a dominant effect, influencing the downstream effects of STAT3 [13].

In contrast to STAT5A, STAT5B has been comparatively understudied, despite mounting evidence of its unique biological functions. For example, STAT5B knockout mice exhibit severe growth retardation and immune dysregulation, phenotypes distinct from those observed in STAT5A-deficient models [8]. In human cancers, STAT5B has been implicated in both promoting and suppressing tumorigenesis, with its effects varying by cancer type and molecular context [14,15]. For instance, STAT5B activation is frequently observed in hematologic malignancies, where it drives proliferation and survival, whereas its downregulation in solid tumors suggests potential tumor-suppressive functions.

This context-dependent duality raises critical questions about whether STAT5B consistently associates with better clinical outcomes or, like STAT5A, exhibits a biphasic role shaped by tissue-specific factors. Furthermore, it remains unclear whether STAT5B can serve as a standalone prognostic marker or whether its utility is contingent upon concurrent analysis of STAT5A and other signaling intermediates. Addressing these gaps is essential for advancing our understanding of STAT5 isoform-specific biology and for developing precision oncology strategies that leverage these insights.

Although STAT5A and STAT5B share approximately 90% amino-acid sequence identity and arise from closely linked genes, they are not functionally redundant. STAT5B contains a unique 20–22-amino-acid C-terminal transactivation extension absent in STAT5A, which enhances DNA-binding affinity, alters transcriptional potency, and modulates interactions with co-regulators [3,6]. These differences underlie distinct physiological roles: STAT5B is the principal mediator of growth-hormone-responsive transcription in hepatocytes and somatic growth [8,9], whereas STAT5A is more tightly linked to prolactin signaling and mammary gland biology [10]. In cancer, STAT5A has been reported to exert context-dependent effects—oncogenic in some hematologic malignancies but tumor-suppressive in breast and other epithelial cancers [5]—whereas STAT5B has been implicated in both oncogenic JAK–STAT signaling in leukemias and potential tumor-suppressive functions in solid tumors [14,15,16,17,18,19]. These structural and functional distinctions highlight the need for isoform-specific analyses to clarify STAT5B’s prognostic significance across malignancies.

**Figure 1:**
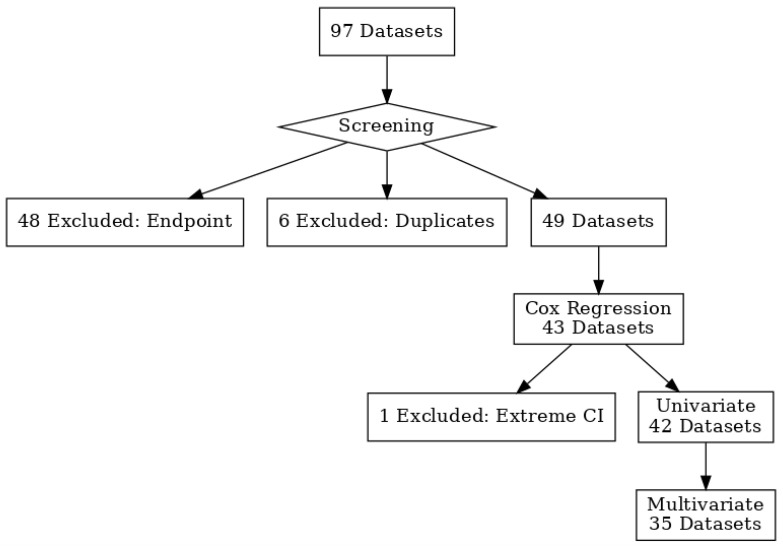
PRISMA 2020 flow diagram illustrating study selection process for STAT5B prognostic analysis. A flowchart illustrating the selection process for datasets used in the survival analysis. Datasets were included based on the availability of overall survival as a clinical endpoint. In cases of duplicate datasets with different probes, the dataset with the smallest log-rank test *p*-value was retained. This filtering process yielded 42 datasets for univariate Cox regression analysis, of which 35 were also used for multivariate analysis based on the availability of individual-level clinical data.

To this end, we conducted a comprehensive meta-analysis of STAT5B expression and survival outcomes across a wide spectrum of cancers, employing the same rigorous bioinformatic pipeline previously applied to STAT5A [5] (Figure 1). Our study leverages publicly available large, uniformly processed microarray datasets with survival annotations to systematically compare the prognostic roles of STAT5A and STAT5B, identify malignancies where STAT5B exerts dominant protective or detrimental effects, and explore the clinical implications of isoform-specific targeting. Microarray data were used because, at the time of analysis, comparably harmonized bulk or single-cell RNA-seq datasets with mature clinical follow-up and consistent probe annotations were not available, whereas the microarray platforms allowed standardized cross-study comparisons. By elucidating the shared and distinct functions of these two isoforms, we aim to provide a foundation for future mechanistic studies and to inform the development of stratified therapeutic approaches that account for STAT5B’s context-dependent roles in cancer.

## 2. Methods

### 2.1. Study Selection Criteria

Publicly available gene-expression datasets were systematically searched using the PrognoScan and GEPIA2 portals. Studies were eligible for inclusion if they (i) were conducted in human subjects; (ii) reported STAT5B (and/or STAT5A) mRNA expression measured on a standardized microarray platform with annotated probe identifiers; (iii) included overall survival or disease-specific survival data with sufficient follow-up to permit Cox modeling; and (iv) had a minimum cohort size of 30 patients. When multiple probes mapped to the same gene, the probe with the smallest log-rank *p*-value was retained for meta-analysis. Datasets were excluded if they lacked survival follow-up, had incomplete or missing clinical annotations for multivariate analysis, or were non-tumor studies (e.g., cell-line experiments). Only one record per independent cohort was retained to avoid duplication. These criteria, and the number of studies excluded at each step, are summarized in the PRISMA flow diagram (Figure 1).

### 2.2. Gene Expression Analysis (GEPIA2)

**Figure 2:**
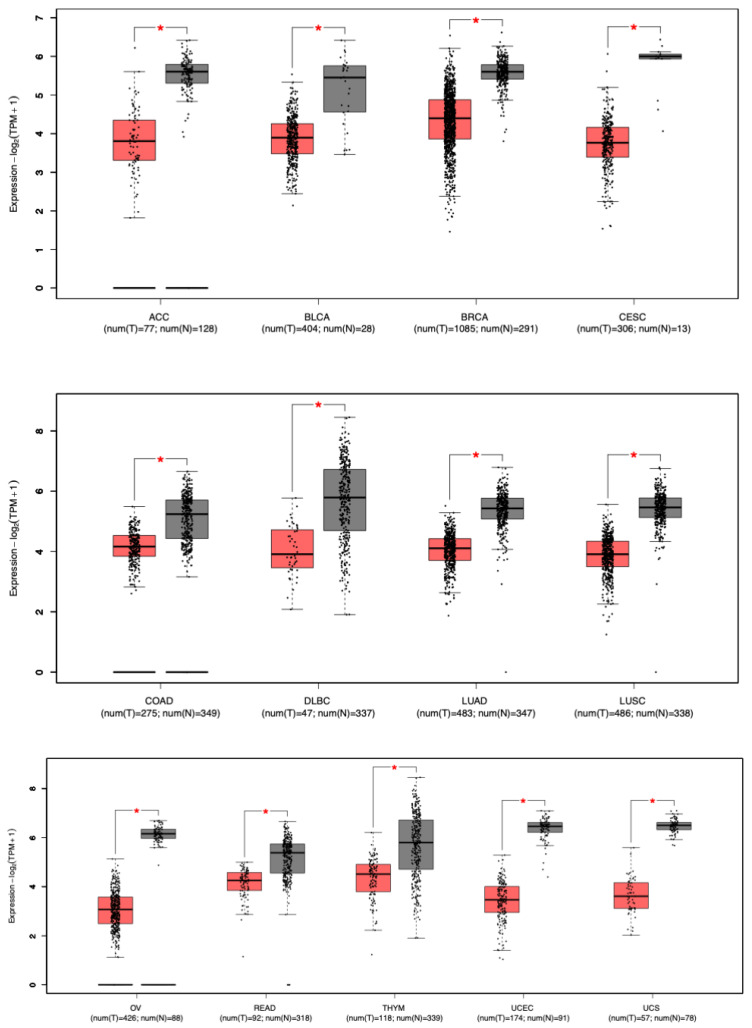
STAT5B mRNA expression in tumor versus normal tissues. Box plots from the GEPIA2 database display STAT5B mRNA expression levels in tumor versus normal tissues. Only cancer types with statistically significant differences are shown. Of the 31 cancer types analyzed, 13 exhibited significantly reduced STAT5B expression in tumors compared to corresponding normal tissues (*p* < 0.01; indicated by *). Red = tumor; black = normal. TPM = transcripts per million; log_2_(TPM + 1) = log-transformed expression values. Cancer type abbreviations: ACC: Adenoid Cystic Carcinoma; BLCA: Bladder Urothelial Carcinoma; BRCA: Breast Invasive Carcinoma; CESC: Cervical Squamous Cell Carcinoma; COAD: Colon Adenocarcinoma; DLBC: Diffuse Large B-cell Lymphoma; GBM: Glioblastoma Multiforme; LUAD: Lung Adenocarcinoma; LUSC: Lung Squamous Cell Carcinoma; OV: Ovarian Serous Cystadenocarcinoma; READ: Rectum Adenocarcinoma; THYM: Thymic Carcinoma; UCEC: Uterine Corpus Endometrial Carcinoma; UCS: Uterine Carcinosarcoma.

The mRNA expression levels of STAT5B were analyzed using the Gene Expression Profiling Interactive Analysis 2 (GEPIA2) platform (http://gepia.cancer-pku.cn/ accessed on 5 January 2022) [16]. This tool integrates RNA-seq data from The Cancer Genome Atlas (TCGA) tumors and Genotype-Tissue Expression (GTEx) normal tissues, enabling a comprehensive comparison of gene expression across 31 cancer types. Differential expression between tumor tissues and their matched normal counterparts was evaluated using One-way ANOVA, with statistical significance determined by applying the Benjamini–Hochberg correction for multiple testing. The thresholds for significance were set at |log_2_FC| > 0.5 and an adjusted *p*-value < 0.01. Results were visualized using Tukey-style boxplots, which display the distribution of transcripts per million (TPM) for STAT5B across the analyzed cohorts (Figure 2).

### 2.3. Survival Analysis (Prognoscan)

The association between STAT5B expression and overall survival was investigated using the Prognoscan database (http://dna00.bio.kyutech.ac.jp/PrognoScan/ accessed on 5 January 2022) [17]. This database aggregates microarray datasets from Gene Expression Omnibus (GEO), ArrayExpress, and independent studies. Datasets were selected based on predefined criteria: only those with overall survival endpoints were included, while duplicates were excluded, retaining probes with the smallest log-rank *p*-value. The final analysis comprised 42 datasets for univariate analysis and 35 datasets for multivariate analysis, the latter including clinical annotations such as age, stage, and grade. Further details are provided in Figure 1 and Table S1.

For statistical processing, the minimum *p*-value approach was employed to dichotomize STAT5B expression into high and low groups. Survival models included univariate Cox regression to estimate hazard ratios (HRs) for STAT5B expression (high vs. low) and multivariate Cox regression to adjust for potential confounders, including age, stage, and grade (see Table S3). Validation was performed using Kaplan–Meier curves, and statistical significance was assessed with log-rank tests.

### 2.4. Meta-Analysis

A meta-analysis was conducted to synthesize the results from multiple datasets. The choice between random-effects and fixed-effects models was guided by heterogeneity metrics: a random-effects model was selected if I^2^ exceeded 50% or if Cochran’s *Q* test yielded a *p*-value < 0.05; otherwise, a fixed-effects model was applied. Heterogeneity was quantified using the I^2^ statistic, and its sources were explored through subgroup analyses stratified by cancer type. Potential publication bias was assessed using funnel plots and Egger’s test, with a *p*-value < 0.05 indicating significant bias. All analyses were performed using R version 4.1.3, leveraging the *survival* package for Cox models and the *meta* package for meta-analysis.

Funnel plot asymmetry was assessed using Egger’s linear regression test. However, we note that this approach is underpowered for subgroups represented by fewer than five datasets, and results for such subgroups should be interpreted with caution. In addition, multivariate Cox regression models were adjusted for age, stage, and grade when available, but other potentially relevant covariates such as mutational status, treatment regimens, and molecular subtype were not consistently reported in the source datasets and therefore could not be incorporated. Interaction analyses (e.g., STAT5B × TP53 mutation status) were not feasible with the available data and remain an important direction for future work.

### 2.5. Assumption Checks

Key assumptions of the Cox proportional hazards model were verified. The proportional hazards assumption was tested using Schoenfeld residuals, and violations were addressed by employing time-stratified models. Outliers were identified and excluded if the width of the 95% confidence interval for the hazard ratio exceeded 10.

### 2.6. Data Quality Control and Bias Mitigation

To minimize biases inherent to publicly available datasets, several quality control measures were implemented. For microarray platforms containing multiple probes for STAT5B, the probe with the smallest log-rank *p*-value was selected to ensure consistency across datasets. Gene expression values from TCGA and GTEx were preprocessed using standardized pipelines provided by GEPIA2, which incorporate batch normalization and cross-platform harmonization to reduce systematic variation between studies. For datasets extracted from Prognoscan, we relied on the database’s standardized preprocessing, which applies quantile normalization across samples to ensure comparability. To control for potential false positives, expression groups (high vs. low) were defined using the minimum *p*-value approach with log-rank testing, and multiple-testing correction (Benjamini–Hochberg method) was applied for expression analyses. These steps collectively aimed to reduce technical noise, enhance reproducibility, and ensure that prognostic associations were not driven by dataset-specific artifacts.

### 2.7. PRISMA Checklist and Reporting Standards

To ensure transparency and reproducibility, the study adhered to the PRISMA (Preferred Reporting Items for Systematic Reviews and Meta-Analyses) guidelines. A completed PRISMA checklist is provided in Table S1, detailing the reporting of each item as recommended by the PRISMA statement. This checklist was referenced during the design and execution of the meta-analysis to ensure comprehensive reporting of methods, results, and conclusions. Additionally, all raw data, analysis scripts, and intermediate files are available in the Supplementary Information to facilitate reproducibility and secondary analyses.

### 2.8. Study Registration and Protocol

The study protocol for data collection and analysis was registered with PROSPERO (International Prospective Register of Systematic Reviews; Registration ID: 1076620) to ensure transparency and minimize reporting bias. The protocol outlined the research objectives, inclusion/exclusion criteria, statistical methods, and planned subgroup analyses. All deviations from the original protocol are explicitly noted in the relevant sections of this manuscript. The registered protocol will be publicly accessible at PROSPERO (https://www.crd.york.ac.uk/PROSPERO/home/ accessed on 5 January 2022).

## 3. Results

### 3.1. STAT5B mRNA Levels Are Significantly Reduced in 42% of Cancer Types

To explore the role of STAT5B in cancer, we compared its mRNA expression levels in tumor versus normal tissues using the GEPIA2 database. Our analysis of STAT5B mRNA expression across 31 cancer types using the GEPIA2 database revealed significant downregulation in 13 cancer types (*p* < 0.01), representing 42% of the studied malignancies (Figure 2). Strikingly, no cancer type exhibited significant upregulation of STAT5B compared to normal tissue. This uniform absence of overexpression, together with the widespread downregulation, underscores STAT5B’s potential role as a broadly conserved tumor-suppressive factor, in contrast to many oncogenes that show tissue-restricted overexpression patterns. The cancers exhibiting the most pronounced reductions included adenoid cystic carcinoma (ACC), bladder cancer (BLCA), breast cancer (BRCA), cervical squamous cell carcinoma (CESC), colon adenocarcinoma (COAD), diffuse large B-cell lymphoma (DLBC), lung adenocarcinoma (LUAD), lung squamous cell carcinoma (LUSC), ovarian cancer (OV), rectum adenocarcinoma (READ), thymic cancer (THYM), uterine corpus endometrial carcinoma (UCEC), and uterine carcinosarcoma (UCS). Notably, no cancer type showed a significant increase in STAT5B expression. These findings suggest a potential tumor-suppressive function for STAT5B across a broad spectrum of cancers. The consistent pattern of downregulation in diverse tumor types implies that STAT5B may play a fundamental role in maintaining normal cellular homeostasis, with its loss contributing to malignant transformation or progression in multiple tissue contexts.

### 3.2. Prognostic Value of STAT5B Expression

**Figure 3:**
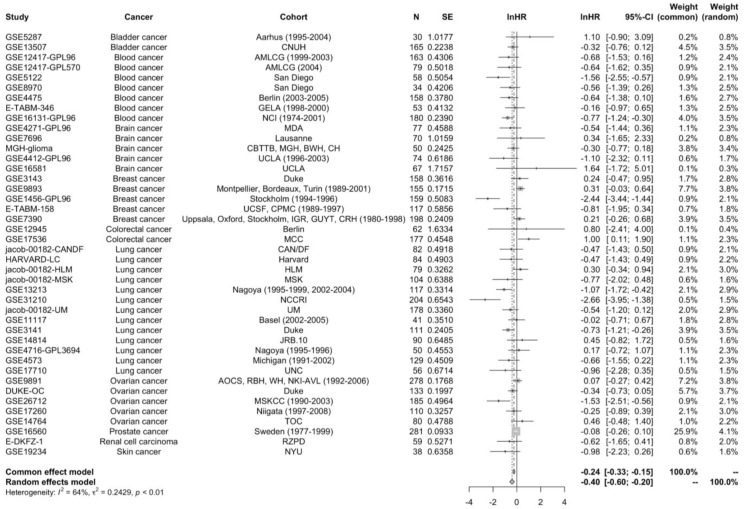
Meta-analysis of hazard ratios for STAT5B expression. Forest plot summarizing hazard ratio estimates from Cox regression analysis across 42 datasets. Random-effects meta-analysis of STAT5B expression and overall survival. Each horizontal line represents a single dataset labeled as “Dataset ID (Cancer type, *n* = sample size)” with its hazard ratio (HR) and 95% confidence interval (CI). HR < 1 indicates improved survival with high STAT5B expression. Squares show the HR estimate for each dataset; horizontal lines denote 95% CI. The red diamond indicates the pooled HR (DerSimonian–Laird random-effects model). I^2^ quantifies between-study heterogeneity. High STAT5B expression was associated with favorable overall survival in cancer patients (lnHR = −0.4009; 95% CI: −0.6007 to −0.2011; *p* < 0.0001). Notable inter-study heterogeneity was observed (I^2^ = 64%; *p* < 0.01).

To assess the prognostic relevance of STAT5B in cancer, we analyzed the relationship between STAT5B expression levels and survival using the Prognoscan database. Survival analysis conducted through the Prognoscan database demonstrated a significant association between high STAT5B expression and improved overall survival. The pooled hazard ratio analysis from 42 datasets revealed a robust protective effect, with high STAT5B expression correlating with better clinical outcomes (lnHR = −0.4009; 95% CI: −0.6007 to −0.2011; z = −3.93; *p* < 0.0001) (Figure 3). This association remained significant after adjusting for key clinical variables in multivariate Cox regression models (Table S3). A random-effects model was used due to significant inter-study heterogeneity (I^2^ = 64%, *p* < 0.01).

The consistency of this finding across multiple independent datasets strengthens the evidence for STAT5B as a favorable prognostic marker. The observed effect size, while modest, was highly statistically significant and maintained its significance even when accounting for inter-study heterogeneity through random-effects modeling. While the effect size (lnHR = −0.4009) is modest, its consistency across multiple datasets suggests a reproducible biological signal. From a clinical standpoint, the modest magnitude indicates that STAT5B expression alone is unlikely to serve as a stand-alone prognostic determinant, but rather may add incremental predictive value when integrated with established biomarkers and clinical variables.

### 3.3. High STAT5B Expression Correlates with Improved Survival in Specific Cancers

**Figure 4:**
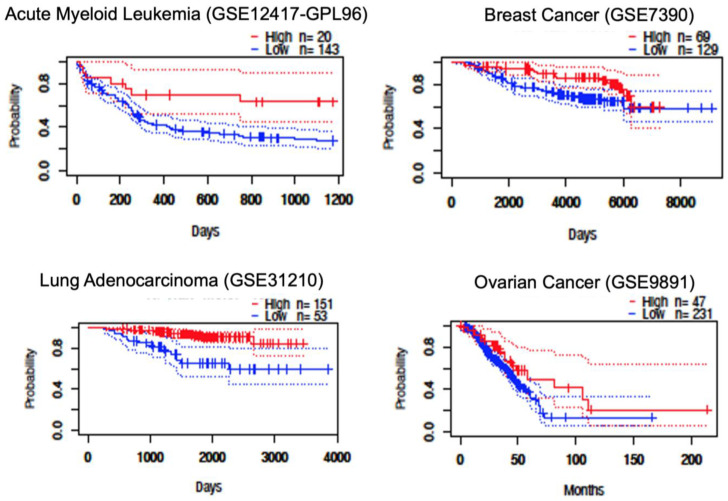
Kaplan–Meier survival analysis by STAT5B expression level. Representative Kaplan–Meier plots comparing overall survival between patients with high versus low STAT5B expression levels across selected cancer types, based on Prognoscan datasets. Favorable survival outcomes were observed in blood, breast, lung, and ovarian cancer patients with high STAT5B expression. See Table S1 for study numbers.

Further examination of cancer-specific patterns revealed particularly strong associations between STAT5B expression and survival outcomes in certain malignancies. In B-cell lymphoma, patients with high STAT5B expression showed markedly prolonged survival compared to those with low expression (Figure 4). Similar trends were observed in lung cancer subtypes, where elevated STAT5B levels correlated with better outcomes across multiple independent cohorts. While breast and ovarian cancers also demonstrated potential survival benefits associated with high STAT5B expression, these associations were less consistent across studies (Figure 4). The variation in effect sizes across cancer types suggests that the prognostic value of STAT5B may depend on tissue-specific molecular contexts or differential roles in various oncogenic pathways.

### 3.4. Subgroup and Meta-Regression Analyses Support Cancer-Specific Effects

**Figure 5:**
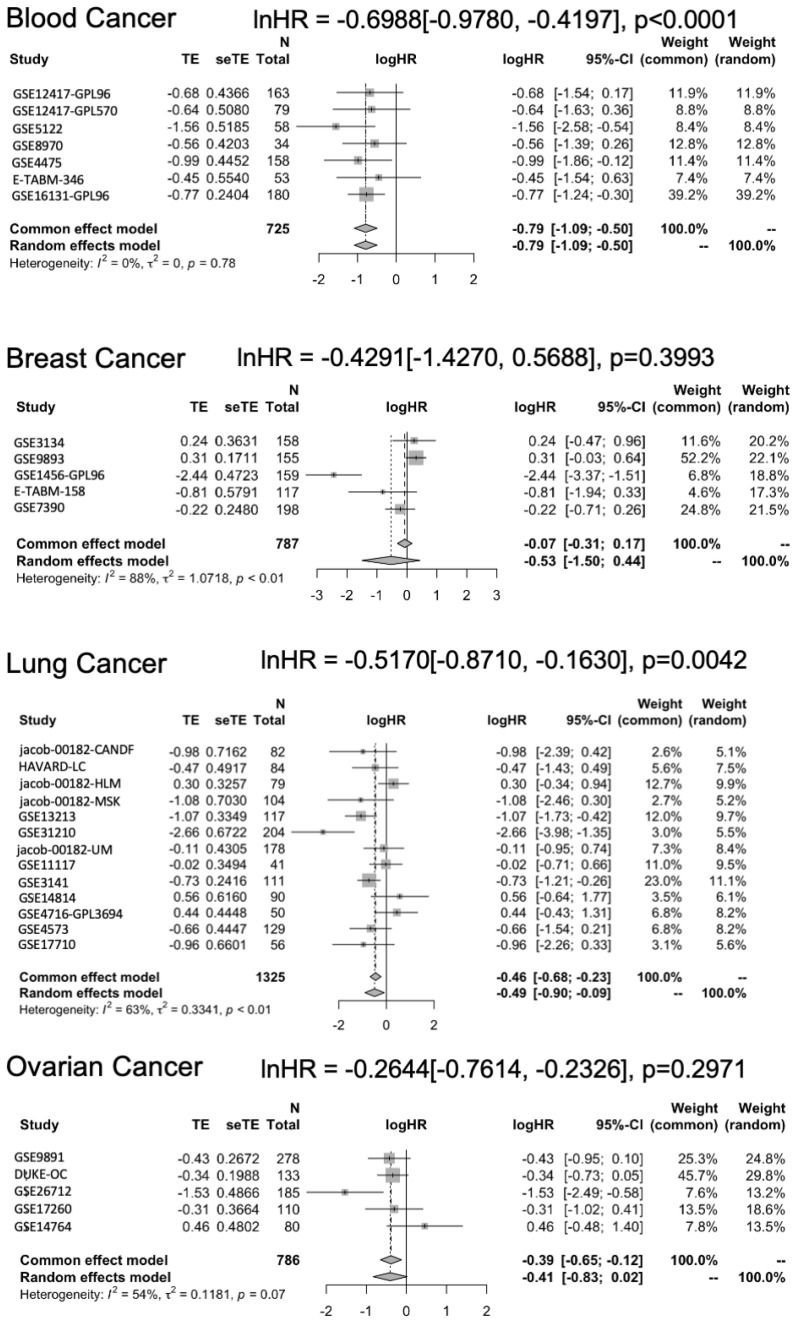
Subgroup meta-analysis by cancer type. Forest plot of unadjusted hazard ratios from subgroup analyses stratified by cancer type. The overall effect estimates and statistical significance are indicated for each subgroup.

To account for the observed heterogeneity, we conducted subgroup and meta-regression analyses. These analyses confirmed that the protective association of high STAT5B expression was particularly strong in hematologic malignancies (lnHR = −0.6988; 95% CI: −0.9780 to −0.4197; *p* < 0.0001) and lung cancers (lnHR = −0.5170; 95% CI: −0.8710 to −0.1630; *p* = 0.0042) (Figure 5). The magnitude of effect in B-cell lymphoma exceeded that seen in other cancer types, potentially reflecting the central role of STAT5 signaling in lymphocyte biology. Meta-regression further supported these findings, with cancer type emerging as a significant modifier of the STAT5B-survival relationship. These cancer-specific patterns offer valuable insights for future research directions and potential clinical applications, suggesting that STAT5B may play particularly relevant roles in specific tumor microenvironments or molecular subtypes.

The protective association of high STAT5B expression was particularly strong in hematologic malignancies, especially in diffuse large B-cell lymphoma. This appears paradoxical given prior reports of oncogenic STAT5B mutations, such as STAT5B^N642H^, which drive proliferation in T-cell leukemias and certain myeloid neoplasms [15,20,21]. One possible explanation is that bulk expression of wild-type STAT5B may reflect preserved differentiation programs or intact chromatin regulatory functions that are favorable for prognosis, whereas oncogenic mutations represent rare, gain-of-function events confined to specific subtypes. Thus, our findings highlight a dichotomy between wild-type STAT5B expression as a potentially protective factor in some hematologic contexts versus mutant or hyperactivated STAT5B as an oncogenic driver. This context-specific duality warrants careful interpretation and emphasizes the need for integrated analyses combining expression with mutational and activation status.

### 3.5. Contextualization with Prior Literature

To situate these findings, we compiled a literature-based summary of cancer-specific roles of STAT5B (oncogenic vs. tumor-suppressive) alongside our meta-analytic results (Table 1). Consistent with our analyses, reports support a tumor-suppressive association in several solid tumors (e.g., lung) and context-dependent protection in breast and ovarian cancer, where we observed downregulation in tumors and favorable survival in subsets [14]. In contrast, hematologic malignancies show divergent roles: STAT5B functions as an oncogenic driver in BCR/ABL-positive leukemia and in T-cell neoplasia harboring activating STAT5B^N642H^ mutations, and is implicated in chronic myeloid neoplasms with eosinophilia/basophilia [15,20,21], whereas our aggregated survival analyses indicate a protective association in B-cell lymphomas. Together, Table 1 highlights the isoform’s strong context dependence and suggests that mutational/activation state and cellular milieu may modulate STAT5B’s prognostic impact [2,3,19].

### 3.6. Assessment of Publication Bias

**Figure 6:**
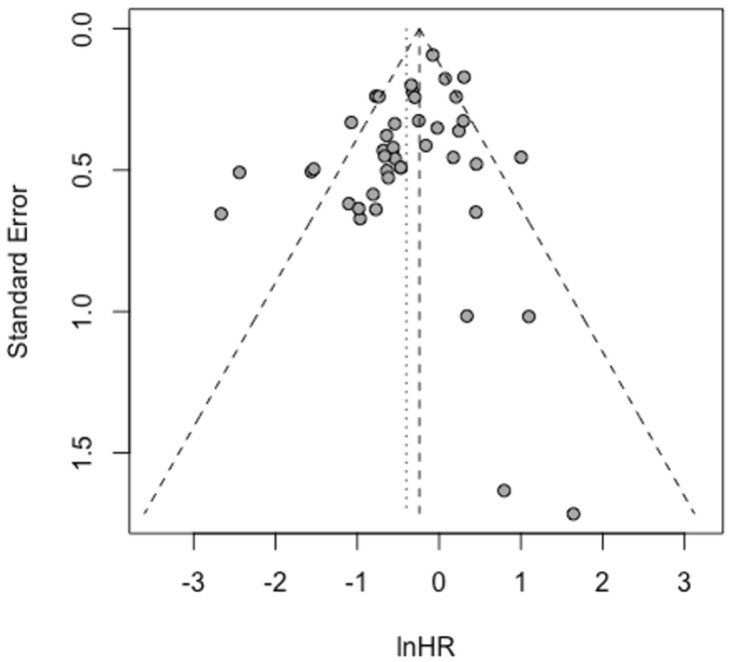
Funnel plot analysis of publication bias. Funnel plot based on adjusted hazard ratios from multivariate Cox regression analysis. Egger’s linear regression test showed no significant evidence of publication bias.

The validity of our meta-analytic findings was supported by comprehensive bias assessment. Funnel plot analysis showed symmetrical distribution of effect sizes, and formal statistical testing using Egger’s regression confirmed the absence of significant publication bias (Egger’s test, *p* = 0.1161) (Figure 6). This rigorous evaluation enhances confidence in the robustness of our conclusions and suggests that the observed associations are unlikely to be artifacts of selective reporting. The consistency of results across different analytical approaches and the lack of detectable bias strengthen the case for STAT5B as a biologically and clinically relevant factor in cancer progression and patient outcomes.

## 4. Discussion

The present study provides systematic evidence that STAT5B plays an important role in cancer biology, with distinct patterns of expression and prognostic impact across tumor types. The frequent downregulation of STAT5B in multiple cancers suggests a tumor-suppressive function, while the consistent association of higher STAT5B expression with improved survival highlights its potential clinical relevance. These associations are cancer-specific, pointing to complex, context-dependent functions that warrant further investigation. In comparison, STAT5A shows favorable prognostic associations in breast cancer but detrimental effects in hematologic malignancies [5], whereas STAT5B appears predominantly tumor-suppressive across epithelial cancers and protective in B-cell lymphomas. Structural differences—such as STAT5B’s unique C-terminal domain—may underlie this divergence [3,6]. Collectively, these findings establish a foundation for future mechanistic studies to define STAT5B’s precise functions and evaluate its potential as a therapeutic target or biomarker.

### 4.1. Dual Roles of STAT5B in Cancer Biology

This meta-analysis reveals a complex landscape in which STAT5B functions largely as a tumor suppressor but retains oncogenic potential in specific contexts. Across 31 cancer types, STAT5B was significantly downregulated in 13 and never upregulated, with higher expression consistently associated with improved survival. These findings suggest that STAT5B may act more broadly as a tumor suppressor than previously recognized, aligning with emerging evidence that unphosphorylated STAT proteins can stabilize heterochromatin and silence oncogenic loci.

Nevertheless, STAT5B’s role in hematologic malignancies appears context-dependent. In leukemias and certain lymphomas, oncogenic activity is often driven by hyperactivation of JAK-STAT signaling or gain-of-function mutations such as STAT5B^N642H^ [14,15,18,19]. This duality parallels STAT3, whose functional output depends on activation state and post-translational modifications [20,21,22,23]. We speculate that STAT5B’s divergent prognostic effects may reflect opposing activities of unphosphorylated STAT5B (uSTAT5B), which maintains chromatin stability, versus phosphorylated STAT5B (pSTAT5B), which drives proliferative signaling. Table 1 summarizes this context dependence and highlights the need for studies integrating STAT5B expression with mutational and signaling profiles [2,3,15,19,20,21].

### 4.2. Tissue-Specific Expression Patterns and Functional Divergence from STAT5A

The pronounced reduction in STAT5B expression in multiple epithelial-derived cancers, including breast, lung, and colorectal tumors, suggests a role in maintaining epithelial homeostasis and suppressing malignant transformation. This contrasts with STAT5A, which displays more variable expression patterns and can act as either oncogenic or tumor-suppressive depending on cellular and molecular context [5,24,25]. Notably, no cancer type analyzed showed STAT5B overexpression, reinforcing its distinct biological and prognostic profile.

Isoform-specific structural differences further underscore their non-redundant functions. STAT5B possesses a unique C-terminal transactivation domain, absent in STAT5A, that influences DNA binding, transcriptional activation potential, and cofactor recruitment [3,6]. This region mediates growth hormone–responsive transcription in hepatocytes [8,9], whereas STAT5A is more closely tied to prolactin signaling and mammary gland biology [10]. These structural distinctions likely underpin the divergent biological behaviors observed: STAT5B exhibits predominantly tumor-suppressive associations across epithelial cancers, while STAT5A demonstrates dual or even opposing effects in certain malignancies [5,19].

Despite their ~90% sequence homology, STAT5A and STAT5B differ markedly in activation profiles and downstream programs. STAT5A is frequently implicated in oncogenic processes, particularly in breast and hematologic cancers [5,19], whereas STAT5B more consistently associates with favorable survival outcomes in epithelial tumors and B-cell lymphomas. These contrasts suggest that isoform-specific differences extend beyond transcriptional targets to include distinct upstream stimuli, post-translational modifications, and protein–protein interactions. Accordingly, comparative analyses of STAT5A and STAT5B in matched tumor contexts are needed to delineate their overlapping yet functionally distinct roles in cancer biology.

### 4.3. Contextualizing STAT5B Findings with STAT5A and Previous Reports

Consistent with our prior systematic analysis of STAT5A [5], the literature supports isoform-specific and tissue-dependent roles for the STAT5 proteins. STAT5A typically shows context-dependent prognostic patterns, favorable outcomes in hormone-responsive breast cancers, but adverse associations in certain hematologic malignancies [14]. In contrast, STAT5B, as confirmed in our meta-analysis, is frequently downregulated in epithelial cancers and associated with improved survival when retained at higher expression levels [14,15,16,17,18,19]. These complementary findings emphasize that STAT5A and STAT5B, despite high sequence homology, exert non-redundant biological effects on tumor behavior and patient prognosis.

Mechanistic differences likely underlie these divergent roles. STAT5B’s unique C-terminal transactivation domain modulates DNA-binding affinity and cofactor interactions [3,6], while STAT5A’s activity aligns more closely with prolactin signaling and mammary gland development [10]. Additionally, differences in upstream signaling, growth hormone-mediated for STAT5B versus prolactin-driven for STAT5A, translate into distinct transcriptional and physiological outcomes [8,9,10].

The apparent discrepancy between STAT5B’s predominantly protective associations in our analysis and prior reports of oncogenic STAT5B mutations (e.g., STAT5B^N642H^) in hematologic malignancies can be reconciled by considering activation state and mutational context. Bulk mRNA expression captured by most microarray datasets primarily reflects wild-type STAT5B, which may mark preserved differentiation or chromatin-stabilizing activity. In contrast, gain-of-function mutations or JAK–STAT hyperactivation produce a qualitatively different, oncogenic signaling state associated with proliferation and poor prognosis [14,15,16,17,18,19]. Thus, our results align with existing literature by emphasizing the context-dependent duality of STAT5B’s role, where its biological outcome depends on expression level, phosphorylation status, and mutational background.

Future integrated analyses combining expression, mutational, and phospho-proteomic data, and directly comparing STAT5A and STAT5B within the same cohorts, will be essential to fully resolve the isoform- and context-specific effects highlighted here.

### 4.4. Prognostic Significance in Hematologic and Solid Tumors

High STAT5B expression was most strongly associated with improved survival in lung adenocarcinomas and B-cell lymphomas, suggesting unique mechanisms by which STAT5B constrains malignant progression. In hematologic cancers, where STAT5B is often considered oncogenic due to its role in cytokine signaling [26,27], our results indicate that tumor-suppressive functions may dominate in specific subtypes. The particularly favorable impact in diffuse large B-cell lymphoma (DLBCL) may reflect STAT5B’s role in preserving B-cell differentiation programs or counteracting oncogenic pathways.

Although our meta-analysis highlights the predominantly tumor-suppressive associations of STAT5B across epithelial cancers and lymphomas, it is equally important to recognize its established oncogenic functions in specific hematologic malignancies. Activating STAT5B mutations, particularly STAT5B^N642H^, have been identified in T-cell large granular lymphocytic leukemia and other hematologic cancers, where they drive constitutive JAK–STAT signaling and malignant proliferation [14,15,18,19]. This apparent contradiction underscores the context-dependent nature of STAT5B biology: while bulk expression of wild-type STAT5B may reflect preserved differentiation or tumor-suppressive chromatin functions, hyperactivated or mutant STAT5B can exert potent oncogenic effects. Overgeneralization of STAT5B as uniformly protective should therefore be avoided, and future studies must integrate expression levels with mutational and phosphorylation status to clarify its role in distinct hematologic subtypes.

### 4.5. Epigenetic Regulation and Non-Canonical Mechanisms

The tumor-suppressive associations of STAT5B are especially intriguing in light of non-canonical roles of STAT proteins in epigenetic regulation. Drosophila STAT maintains heterochromatin through HP1 recruitment [28,29], and human uSTAT5A and uSTAT3 similarly stabilize heterochromatin and suppress oncogenic transcription [30,31]. Our finding that STAT5B’s strongest survival associations occur in cancers with frequent epigenetic dysregulation (e.g., DLBCL, LUAD) raises the hypothesis that uSTAT5B contributes to heterochromatin stability. While parallels with STAT5A and STAT3 support this possibility [22,23,24,25], direct experimental validation—for example, through ChIP-seq or chromatin interaction assays—is required.

### 4.6. Clinical Implications and Therapeutic Potential

The consistent association of high STAT5B expression with favorable survival across independent datasets highlights its promise as a prognostic biomarker. In lung cancer and DLBCL, STAT5B expression could complement existing markers to refine risk stratification and guide treatment intensity. Moreover, the distinct biological roles of STAT5 isoforms have therapeutic implications. STAT5 inhibitors are under development for hematologic cancers, yet our findings suggest that indiscriminate inhibition of STAT5 activity may inadvertently suppress STAT5B’s tumor-suppressive functions in solid tumors. Isoform-specific modulation of STAT5 activity may therefore be a more effective strategy for precision oncology.

## 5. Limitations

This study has several limitations. First, mRNA expression does not always reflect protein abundance or phosphorylation status, and future studies should validate findings at the protein level using immunostaining or proteomics. Second, the molecular mechanisms underlying STAT5B’s tumor-suppressive effects remain to be fully defined, particularly whether they involve canonical transcriptional programs, non-canonical chromatin regulation, or both. Third, tissue-specific variability in STAT5B’s prognostic effects may arise from differences in cofactor interactions, downstream targets, or post-translational modifications.

Analytical limitations also warrant consideration. Funnel plots and Egger’s tests are underpowered in subgroups with few datasets, and absence of bias should be interpreted cautiously. While multivariate analyses adjusted for age, stage, and grade, unmeasured factors such as mutation status (e.g., STAT5B^N642H^, TP53), treatment regimens, and molecular subtype could not be controlled. Interaction analyses were not feasible with the available data but remain an important area for future research.

### 5.1. Future Directions

Future research should integrate STAT5B expression with mutational profiling (e.g., STAT5B^N642H^, TP53) to better distinguish oncogenic from tumor-suppressive contexts, while also validating findings at the protein level, including phosphorylation status, to clarify its prognostic significance. Functional studies such as ChIP-seq and cofactor interaction assays will be essential to test the hypothesis that unphosphorylated STAT5B contributes to heterochromatin stability. In parallel, direct comparative analyses of STAT5A and STAT5B within the same tumor types are needed to disentangle their overlapping and divergent functions, ultimately enabling the development of isoform-specific diagnostic tools and therapeutic strategies.

### 5.2. Concluding Remarks

This comprehensive analysis identifies STAT5B as a multifaceted regulator of cancer biology, with predominantly tumor-suppressive characteristics across epithelial cancers and protective associations in lymphomas, but oncogenic potential in specific hematologic contexts. The findings expand understanding of STAT5B beyond its established role in leukemias, highlighting a broader tumor-suppressive function. The striking divergence between STAT5A and STAT5B underscores the necessity of isoform-specific analyses in both research and clinical applications. Together, these insights provide a framework for future mechanistic and translational studies and suggest that STAT5B may represent both a biomarker and a therapeutic target in precision oncology.

## Figures and Tables

**Table 1:** Reported cancer-specific roles of STAT5B as tumor suppressor or oncogene.

Cancer Type	Role of STAT5B	Findings	Representative References
B-cell lymphoma (DLBCL, others)	Tumor suppressor (protective)	High STAT5B expression associated with favorable overall survival in our meta-analysis. May contribute to maintaining B-cell differentiation programs.	This study; Maurer et al., 2019 [3]; Kollmann et al., 2019 [2]
T-cell neoplasia	Oncogene	Activating mutation STAT5B^N642H identified as driver mutation in T-cell acute lymphoblastic leukemia; promotes proliferation.	Pham et al., 2018 [20]
Chronic myeloid neoplasms (with eosinophilia/basophilia)	Oncogene	STAT5B mutations reported in myeloid malignancies; linked to disease progression.	Yin et al., 2024 [21]
Leukemia (BCR-ABL positive)	Oncogene	STAT5B required for maintenance of BCR/ABL-driven leukemia.	Hoelbl et al., 2010 [15]
Breast cancer	Tumor suppressor/mixed	Downregulation of STAT5B observed in tumor tissue; high STAT5B expression associated with improved survival in our analysis. However, STAT5 signaling can also promote survival under certain contexts.	Yamashita et al., 2006 [14]; This study
Lung adenocarcinoma/squamous carcinoma	Tumor suppressor	High STAT5B expression correlated with favorable prognosis; frequent downregulation observed in tumor tissue.	This study
Ovarian cancer	Tumor suppressor (context-dependent)	High STAT5B expression associated with better survival in some datasets, though results inconsistent due to limited sample size.	This study
Colon adenocarcinoma	Tumor suppressor	STAT5B expression significantly reduced in tumors vs. normal tissue. Prognostic value less clear due to limited data.	This study
Prostate cancer	Oncogene (reported)	STAT5B activity reported to promote growth in androgen receptor–positive prostate cancer.	Ferbeyre & Moriggl, 2011 [19]

Classification as “oncogene” or “tumor suppressor” is based on reported biological activity or clinical associations. Where conflicting results exist, the table notes “context-dependent” effects.

## Data Availability

All supporting data are included within the main article and its Supplementary files.

## Supplementary Materials

The following supporting information can be downloaded at: https://www.mdpi.com/article/10.3390/biom15111503/s1, Table S1: PRISMA Checklist; Table S2: Dataset information from Prognoscan database; Table S3: Covariate information of datasets used in multivariate Cox regression analysis.

## Abbreviations

ACC	Adenoid Cystic Carcinoma
BLCA	Bladder Urothelial Carcinoma
BRCA	Breast Invasive Carcinoma
CESC	Cervical Squamous Cell Carcinoma
CI	Confidence Interval
COAD	Colon Adenocarcinoma
DLBC	Diffuse Large B-cell Lymphoma
GAS	γ-interferon activation sites
GBM	Glioblastoma Multiforme
GEPIA	Gene Expression Profiling Interactive Analysis
GEO	Gene Expression Omnibus
GTEx	Genotype-Tissue Expression
HR	Hazard Ratio
LUAD	Lung Adenocarcinoma
LUSC	Lung Squamous Cell Carcinoma
OV	Ovarian Serous Cystadenocarcinoma
PRISMA	Preferred Reporting Items for Systematic Reviews and Meta-Analyses
PROSPERO	Prospective Register of Systematic Reviews
READ	Rectum Adenocarcinoma
STAT	Signal Transducer and Activator of Transcription
TCGA	The Cancer Genome Atlas
THYM	Thymic Carcinoma
UCEC	Uterine Corpus Endometrial Carcinoma
UCS	Uterine Carcinosarcoma
