# Supplementary material for: The Prognostic Role of STAT5B Across Cancer Types and Comparative Analysis with STAT5A: A Systematic Review
Source: Biomolecules. 2025 Oct 24;15(11):1503. doi: 10.3390/biom15111503 (PMC12649934; doi:10.3390/biom15111503)
Supplement: Supplementary file 1 [file biomolecules-15-01503-s001.zip › Table S1&3.pdf]

Supplementary Information

The Prognostic Role of STAT5B Across Cancer Types and Comparative Analysis with STAT5A: A Systematic Review

Christine Maninang, Jinghong Li, and Willis X. Li

Department of Medicine
University of California San Diego
La Jolla, CA, United States

* Corresponding author: Willis X. Li (wxli@health.ucsd.edu)

Table S1. PRISMA Checklist

Section and Topic	Item #	Checklist item	Location where item is reported
		**TITLE**	
Title	1	Identify the report as a systematic review.	Title page
		**ABSTRACT**	
Abstract	2	Structured summary.	Abstract
		**INTRODUCTION**	
Rationale	3	Describe the rationale for the review in the context of existing knowledge.	Introduction, para 1-3
Objectives	4	Provide an explicit statement of the objective(s) or question(s) the review addresses.	Introduction, final para
		**METHODS**	
Eligibility criteria	5	Specify the inclusion and exclusion criteria for the review and how studies were grouped for the syntheses.	Methods: "Datasets were selected..."
Information sources	6	Specify all databases, registers, websites, organisations, reference lists and other sources searched or consulted to identify studies. Specify the date when each source was last searched or consulted.	Methods: GEPIA2/Prognoscan
Search strategy	7	Present the full search strategies for all databases, registers and websites, including any filters and limits used.	Methods: Database parameters. For microarray platforms with multiple probes for STAT5B, the probe with the smallest log-rank p-value was retained.
Selection process	8	Specify the methods used to decide whether a study met the inclusion criteria of the review, including how many reviewers screened each record and each report retrieved, whether they worked independently, and if applicable, details of automation tools used in the process.	Methods: Inclusion/exclusion. Datasets lacking overall survival endpoints or with incomplete probe annotation were excluded.
Data collection process	9	Specify the methods used to collect data from reports, including how many reviewers collected data from each report, whether they worked independently, any processes for obtaining or confirming data from study investigators, and if applicable, details of automation tools used in the process.	Methods: Statistical processing. Datasets with incomplete clinical annotations (e.g., age, stage, grade) were excluded from multivariate analyses.
Data items	10a	List and define all outcomes for which data were sought. Specify whether all results that were compatible with each outcome domain in each study were sought (e.g. for all measures, time points, analyses), and if not, the methods used to decide which results to collect.	Methods: Survival analysis
	10b	List and define all other variables for which data were sought (e.g. participant and intervention characteristics, funding sources). Describe any assumptions made about any missing or unclear information.	Methods: Survival analysis. Missing clinical covariates were documented; datasets with major missing information were excluded from multivariate analysis.

Section and Topic	Item #	Checklist item	Location where item is reported
Study risk of bias assessment	11	Specify the methods used to assess risk of bias in the included studies, including details of the tool(s) used, how many reviewers assessed each study and whether they worked independently, and if applicable, details of automation tools used in the process.	Methods: Assumption checks
Effect measures	12	Specify for each outcome the effect measure(s) (e.g. risk ratio, mean difference) used in the synthesis or presentation of results.	Methods: HR calculations
Synthesis methods	13a	Describe the processes used to decide which studies were eligible for each synthesis (e.g. tabulating the study intervention characteristics and comparing against the planned groups for each synthesis (item #5)).	Methods: Meta-analysis
	13b	Describe any methods required to prepare the data for presentation or synthesis, such as handling of missing summary statistics, or data conversions.	Methods: Meta-analysis
	13c	Describe any methods used to tabulate or visually display results of individual studies and syntheses.	Methods: Meta-analysis
	13d	Describe any methods used to synthesize results and provide a rationale for the choice(s). If meta-analysis was performed, describe the model(s), method(s) to identify the presence and extent of statistical heterogeneity, and software package(s) used.	Methods: Meta-analysis
	13e	Describe any methods used to explore possible causes of heterogeneity among study results (e.g. subgroup analysis, meta-regression).	Methods: Meta-analysis
	13f	Describe any sensitivity analyses conducted to assess robustness of the synthesized results.	Methods: Meta-analysis
Reporting bias assessment	14	Describe any methods used to assess risk of bias due to missing results in a synthesis (arising from reporting biases).	Methods: Funnel plots/Egger's
Certainty assessment	15	Describe any methods used to assess certainty (or confidence) in the body of evidence for an outcome.	Results: Subgroup analyses
**RESULTS**			
Study selection	16a	Describe the results of the search and selection process, from the number of records identified in the search to the number of studies included in the review, ideally using a flow diagram.	Results: First paragraph
	16b	Cite studies that might appear to meet the inclusion criteria, but which were excluded, and explain why they were excluded.	Results: First paragraph
Study characteristics	17	Cite each included study and present its characteristics.	Table S1
Risk of bias in studies	18	Present assessments of risk of bias for each included study.	Results: Heterogeneity
Results of individual studies	19	For all outcomes, present, for each study: (a) summary statistics for each group (where appropriate) and (b) an effect estimate and its precision (e.g. confidence/credible interval), ideally using structured tables or plots.	Figure 3
Results of syntheses	20a	For each synthesis, briefly summarise the characteristics and risk of bias among contributing studies.	Results: Meta-analysis
	20b	Present results of all statistical syntheses conducted. If meta-analysis was done, present for each the summary estimate and its precision (e.g. confidence/credible interval) and measures of statistical heterogeneity. If comparing groups, describe the direction of the effect.	Results: Meta-analysis
	20c	Present results of all investigations of possible causes of heterogeneity among study results.	Results: Meta-analysis
	20d	Present results of all sensitivity analyses conducted to assess the robustness of the synthesized results.	Results: Meta-analysis

Section and Topic	Item #	Checklist item	Location where item is reported
Reporting biases	21	Present assessments of risk of bias due to missing results (arising from reporting biases) for each synthesis assessed.	Results: Publication bias
Certainty of evidence	22	Present assessments of certainty (or confidence) in the body of evidence for each outcome assessed.	Discussion: Limitations
**DISCUSSION**			
Discussion	23a	Provide a general interpretation of the results in the context of other evidence.	Discussion
	23b	Discuss any limitations of the evidence included in the review.	Discussion
	23c	Discuss any limitations of the review processes used.	Discussion
	23d	Discuss implications of the results for practice, policy, and future research.	Discussion
**OTHER INFORMATION**			
Registration and protocol	24a	Provide registration information for the review, including register name and registration number, or state that the review was not registered.	Methods: PROSPERO
	24b	Indicate where the review protocol can be accessed, or state that a protocol was not prepared.	Methods: PROSPERO
	24c	Describe and explain any amendments to information provided at registration or in the protocol.	Methods: PROSPERO
Support	25	Describe sources of financial or non-financial support for the review, and the role of the funders or sponsors in the review.	Acknowledgments
Competing interests	26	Declare any competing interests of review authors.	Title page
Availability of data, code and other materials	27	Report which of the following are publicly available and where they can be found: template data collection forms; data extracted from included studies; data used for all analyses; analytic code; any other materials used in the review.	Methods: OSF repository

Additional Notes

PRISMA Flow Diagram: Included as **Figure 1** (study selection process).

Supplementary Tables: Table S2 (Dataset information from Prognoscan database), **Table S3** (Covariate information of datasets used in multivariate Cox regression analysis).

Software: R packages (*survival*, *meta*) cited in Methods.

Table S3. Covariate information of datasets used in multivariate Cox regression analysis

Dataset	Cancer	Age (Years) Range (Median)	Sex	Grade	Stage	Other Clinical Characteristics
GSE13507	Bladder	24 – 88 (66)	F: 18.2% M: 81.8%	Low: 63.6%. High: 36.4%		Therapy: systematic chemotherapy: 16.4%
GSE12417-GPL96	Hematological	17-83(58)				
GSE12417-GPL570	Hematological	18-85(62)				
GSE5122	Hematological	19-85(63)	F: 51.7% M: 48.3%			Tipifarnib best response: CR: 5.17 % PR: 1.72% hematologic improvement: 10.34 % SD: 6.90% NR: 75.86%
GSE8970	Hematological	63-85(73)	F: 32.4% M: 67.6%			Attribute: organ dysfunction: 55.9% Attribute: prior myelodysplastic syndromes (MDS): 73.5% Tipifarnib response: CR: 26.5%. hematological improvement: 11.8% PR: 38.2% SD: 23.5%
GSE4475	Hematological	2-93(63)	F: 43% M: 57%			
E-TABM-346	Hematological	59 – 79(69)	F: 49.1% M: 50.9%	2: 18.9% 3: 49.1% 4: 17.0% 5: 15.1%		
GSE4271-GPL96	Brain	22-82(45)	F: 32.5% M: 67.5%	3: 27.3% 4: 72.7%		
GSE7696	Brain	26.4-70.3 (52.2)	F: 27.1% M: 72.9%	_		
GSE4412-GPL96	Brain	18-82(42.5)	F: 62.2% M: 37.8%	3: 32.4% 4: 67.6%		
GSE9893	Breast cancer	42.6 – 92.4 (68.2)				Therapy: adjuvant therapy: TAM: 21.9% X-RAY+TAM: 73.5% X-RAY+TAM+LHRH: 4.5%
E-TABM-158	Breast cancer					Positive for ER marker: 63.2% N score: N0: 43.6% N1: 56.4%

GSE7390	Breast cancer	24 – 60(46)				Positive for ER marker: 67.7%<br>Tumor size range: 0.6 – 5.0 cm (median: 2 cm)
GSE12945	Colorectal cancer	38 – 87(65)	0: 45.2%<br>1: 54.8%	2:50%<br>3:50%	I: 21.0%<br>II: 37.1%<br>III: 33.9%<br>IV: 8.1%	
GSE17536	Colorectal cancer	26- 92(66)	F: 47.6%<br>M: 52.4%	1: 10.3%<br>2: 76.6%<br>3: 13.1%	I: 16.6%<br>II: 37.9%<br>III: 38.6%<br>IV: 6.9%	Ethnicity:<br>Caucasian: 85.3%<br>Black: 5.1%<br>Hispanic: 0.6%<br>Other: 9.0%
GSE17537	Colorectal cancer	23- 94(62)	F: 52.7%<br>M: 47.3%		I: 7.3%<br>II: 27.3%<br>III: 34.5%<br>IV: 30.9%	
jacob-00182-CANDF	Lung cancer	35- 79 (61)	F: 43.9%<br>M: 56.1%			N score: N0: 70.7%. N1: 29.3%<br>T score: T1: 20.7% T2: 76.8% T3: 2.4%
HARVARD-LC	Lung cancer	33-88(64)	F: 57.1%<br>M: 42.9%		I: 74%<br>II: 17%<br>III: 9%	
jacob-00182-HLM	Lung cancer	36- 87(68)	F: 49.4%<br>M: 50.6%			
MICHIGAN-LC	Lung cancer	40.9-84.6(63.7)	F: 59.3%<br>M: 40.7%		I: 77.9%<br>III: 22.1%	
jacob-00182-MSK	Lung cancer	38-82(65)	F: 64.4%<br>M: 35.6%			N score: N0: 61.5% N1: 19.2% N2: 19.2%<br>T score: T1: 31.7% T2: 64.4%. T3: 3.8%
GSE13213	Lung cancer	38- 84(61)	F: 48.7%<br>M: 51.3%		I: 56%<br>II: 15%<br>III: 29%	KRAS mutation status: mutant: 2.8% wildtype: 87.2%
GSE31210	Lung cancer	30-76(61)	F: 53.4%<br>M: 46.6%		I: 79.4%<br>II: 20.6%	Marker MYC:<br>high: 7.8%. low: 91.7%. not detected: 0.5%<br>Smoking status: ever-smoker: 48.5%. never-smoker: 51.5%<br>Gene alteration status: ALK-fusion +: 3.4%<br>EGFR mutation + :56.9%<br>EGFR/KRAS/ALK -: 30.4%<br>KRAS mutation +: 9.3%

jacob-00182-UM	Lung cancer	33-86(65)	F: 43.3%<br>M: 56.7%			N score: N0: 71.9% N1: 16.3% N2: 11.8%<br>T score: T1: 49.4% T2: 39.3%. T3: 7.3% T4:3.9%
GSE11117	Lung cancer	42-80(66)	F: 34.1%<br>M: 65.9%		I: 17.1%<br>II: 19.5%<br>III: 21.9%<br>IV: 41.5%	
GSE14814	Lung cancer	38.2-81.3(63.3)	F: 25.6%<br>M: 74.4%		I: 50%<br>II: 50%	
GSE4573	Lung cancer	42-91(68)	F: 36.4%<br>M: 63.6%		I: 56%<br>II: 26%<br>III: 18%	
GSE17710	Lung cancer	41-85(67)	F: 42.9%<br>M: 57.1%	Mod: 64.3%<br>Poor: 35.7%	I: 60.7%<br>II: 34%<br>III: 5.4%	
GSE9891	Ovarian cancer	22-80(59)				
GSE8841	Ovarian cancer	21.1-87.1(49.9)		1: 16.0%<br>2: 24.7%<br>3: 40.7%<br>Borderline: 18.5%	1A: 30.9%<br>1B: 6.2%<br>1C: 63%	
GSE26712	Ovarian cancer	-				Attribute: cytoreductive surgery outcome: optimal: 48.6% suboptimal: 51.4%
GSE17260	Ovarian cancer				III: 84.6%<br>IV: 15.5%	Attribute: cytoreductive surgery outcome: not optimal: 48.2% optimal: 51.8%
GSE14764	Ovarian cancer			1: 3.8%<br>2: 28.7%<br>3: 67.5%	I: 10%<br>II: 1.2%<br>III: 86.2%<br>IV: 2.5%	
GSE16560	Prostate cancer	51-91(74)				Gleason score range: 6-10<br>Median: 7
GSE19234	Skin cancer	30-92(62.5)	F: 36.8%<br>M: 63.2%		III: 86.8%<br>IV: 13.2%	

F = female, M= male, ER= estrogen receptor, KRAS= Kirsten rat sarcoma virus gene, EGFR= epidermal growth factor receptor, ALK= anaplastic lymphoma kinase, TAM= tamoxifen, LHRH= luteinizing-hormone-releasing hormone agonists
